# The minimally invasive transventricular endoscopic approach to third ventricular lesions in pediatric patients—all-rounder with limitations?

**DOI:** 10.1007/s00381-023-06096-8

**Published:** 2023-07-29

**Authors:** Fritz Teping, Joachim Oertel

**Affiliations:** Department of Neurosurgery, Faculty of Medicine, Saarland University, Kirrbergerstraße, Building 90.5, D-66421 Homburg, Germany

**Keywords:** Pediatric, Neuroendoscopy, Ventricular surgery, Endoscopy

## Abstract

**Introduction:**

The surgical management of third ventricular lesions poses unique challenges, requiring careful consideration of various approaches and techniques. This study focuses on the transventricular transforaminal endoscopic approach and aims to provide insights into its indications, limitations, technical nuances, and potential complications in pediatric patients.

**Methods:**

A retrospective analysis was conducted using data from a 13-year period on pediatric patients who were subjected to transforaminal endoscopic surgery for third ventricular lesions. The study utilized a prospectively maintained internal database, extracting demographic data, preoperative assessment, surgical details, and postoperative follow-up information. The surgical technique is presented in detail, and exemplary case reports highlight relevant surgical considerations.

**Results:**

Out of 578 endoscopic transforaminal procedures, 24 surgeries were performed on pediatric patients with third ventricular lesions. Performed procedures consisted of cyst resection (13 cases), solid tumor resection (4 cases), and tumor biopsies with CSF pathway restoration (7 cases). The mean age at the time of surgery was 7.6 years. Postoperatively, 14 patients showed transient nausea and vomiting (58.3%); 10 patients showed pneumocephalus on postoperative MRI (41.7%). No emergency postoperative re-interventions nor perioperative mortality were observed.

**Conclusion:**

The endoscopic transventricular transforaminal approach is a safe approach for lesion resection, CSF pathway restoration, and tumor biopsy in pediatric patients with third ventricle lesions. The author’s results support the use of this minimally invasive technique as an alternative to more extensive approaches, particularly to the interforniceal interhemispheric approach. However, surgical success is highly dependent to the individual surgeon’s experience and moreover to a suitable indication setting. Careful preoperative planning and knowledge of the approaches’ pro and cons is mandatory for successful application of this approach.

## Introduction

Surgery of lesions of the third ventricle remains one of the most challenging tasks in intracranial surgery. The third ventricle is not only one of the few deep-seated locations in the center of the brain; it is also surrounded by numerous highly vulnerable structures which are not to be harmed at any costs during surgery. Finally, no single “work horse” approach to the third ventricle exists which enables its application in most of the indications. Thus, a high number of different approaches are applied with highly various indications and reported complications rates. Achieving safe and effective surgical outcomes requires meticulous planning, considering the precise location and configuration of the lesion and the personal abilities of the performing surgeon. Further to these considerations, various techniques of visualization have been proposed, including microscopic or endoscopic visualization, as well as combinations of both or even more recently exoscopic techniques.

Common choices for accessing the third ventricle include the transcallosal interforniceal approach [1–4], the subfrontal translamina terminalis approach [2, 5–7], the transventricular transforaminal approach [2, 8–11], the transventricular transchoroidal approach, the supracerebellar-infratentorial approach [12–15], and the interhemispheric transtentorial occipital approach. Each of these approaches has been presented with several variations. Different pathological entities, such as tumors and cysts, in combinations of the location of the lesion within the third ventricle require individualized surgical strategies, ranging from biopsy-only procedures to gross-total resections and restoration of cerebrospinal fluid (CSF) pathways in cases of occlusive hydrocephalus. Irrespective of the chosen method, it is crucial to minimize surgical trauma and brain retraction, while enabling best possible visualization and surgical control to respond adequately and immediately to any unexpected incidents during the procedure (Table 1).

**Table 1:** Surgical entry points for endoscopic procedures within the third ventricle

**Lesion location**	**Burr hole placement**
Foramen of Monro	3–5 cm parasagittal—2–4 cm precoronal
Anterior part of the third ventricle	1–2 cm parasagittal—coronal
Posterior part of the third ventricle	1–2 cm parasagittal—4–6 cm precoronal
Aqueduct and fourth ventricle	1–2 cm parasagittal—3–6 cm precoronal (combined)

The pure endoscopic transventricular transforaminal approach, utilizing a precoronal burr hole, has emerged as the gold standard for cerebrospinal fluid (CSF) pathway restoration via endoscopic third ventriculostomy (ETV) in adults and children [16–20]. Additionally, this approach has demonstrated efficacy in the treatment of various lesions within the third ventricle, including colloid cysts and certain tumor entities [9, 21–26]. However, the successful execution of these procedures relies heavily on the expertise of the surgeon and the appropriateness of the surgical planning and goals (Table 2).

**Table 2:** General information on the study population

Characteristics		Procedures (*n* = 24)
Male: female		13: 11
Age at surgery		7.6 (± 6.1) years
Pathology	Cystic lesion	15 (%)
	Tumor	9 (37.5%)
Procedure	Cyst fenestration	14 (58.3%)
	Cyst resection	2 (8.3%)
	Tumor biopsy	4 (16.7%)
	Tumor resection	4 (16.7%)
Additional CSF pathway restoration	ETV	9 (37.5%)
	Catheter placement	11 (45.8%)
Premature termination of surgery		0 (0%)
Postoperative pneumocephalus		10 (41.7%)
Surgical mortality		0 (0%)

In this study, the authors present their experiences with the transventricular transforaminal endoscopic approach for third ventricular lesions over a period of 13 years. It is the purpose to demonstrate suitable indications, limitations, technical nuances, and potential complications associated with this surgical technique. By doing so, the presented study should contribute to the existing body of knowledge on the management of third ventricular lesions, highlighting the role of the endoscopic transventricular approach in comparison to other available approaches.

## Methods

### Data acquisition and processing

A retrospective analysis was conducted using data obtained from a prospectively maintained internal database. The database consisted of medical records, operative reports, and radiological imaging findings of patients who underwent the transventricular transforaminal endoscopic approach for third ventricular lesions over a 13-year period. All data of procedures from December 2010 to June 2013 were included. Only patients who were below the age of 18 at time of surgery, suffering from a third ventricular lesion, were included in the final analysis. The data were collected from the Department of Neurosurgery, Saarland University Medical Center only. Patient information, including demographic data, preoperative assessment, surgical details, and postoperative follow-up, was extracted from the database for analysis. All patient data were de-identified and handled in accordance with relevant privacy regulations and institutional guidelines. All data were managed using SPSS (IBM Corp., Armonk, NY, USA).

### Statistical analysis

Due to the relatively small number of participants in this study, the statistical analysis focused on descriptive statistics. The emphasis of this study is on providing a detailed description of the surgical technique and presenting the findings in a descriptive manner. Descriptive statistics were computed to summarize patient characteristics, surgical outcomes, and clinical follow-up data. Measures such as means, standard deviations, and frequency distributions were used to provide a comprehensive overview of the study population.

### Surgical technique

**Fig. 1:**
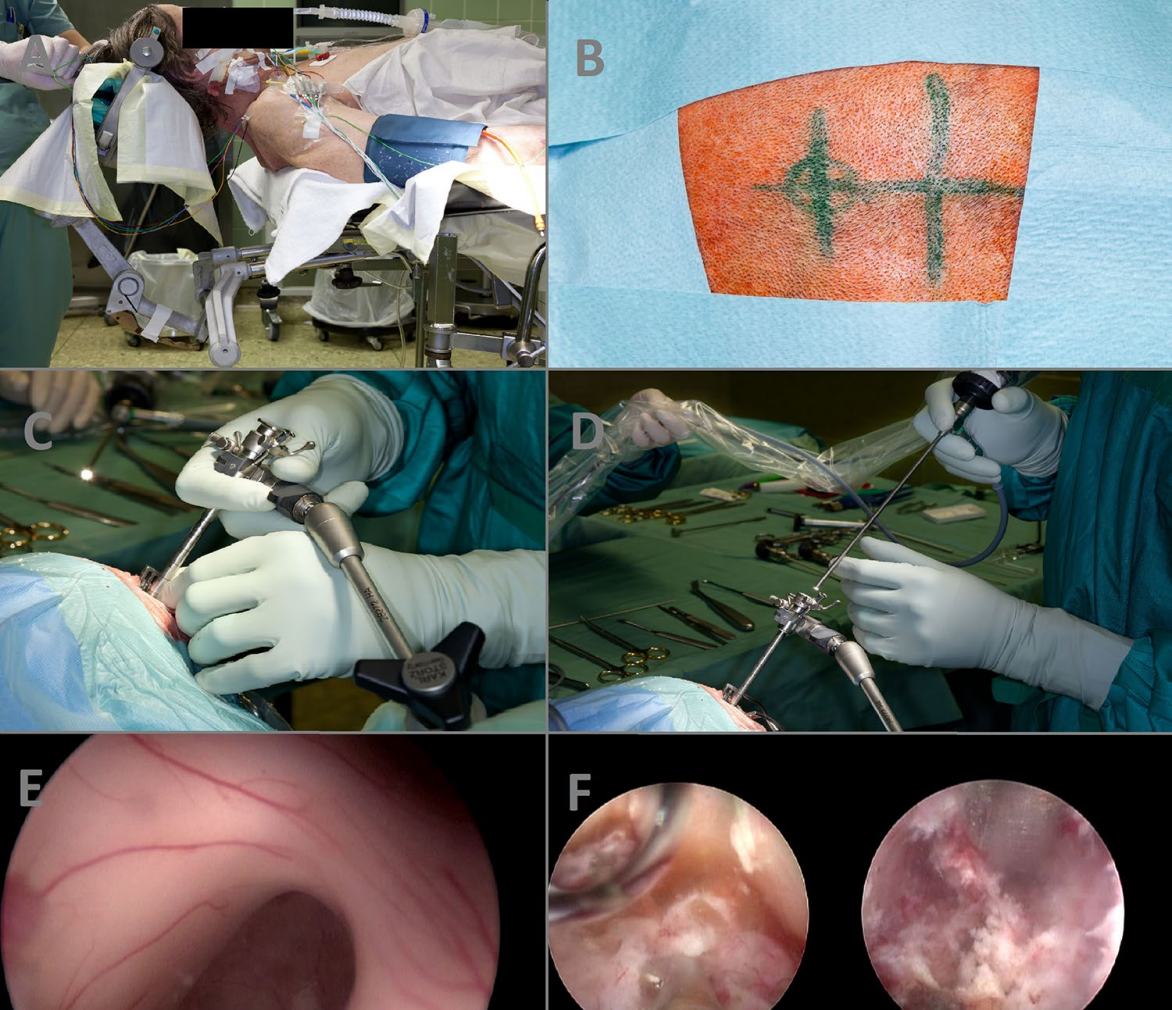
**A** Patient positioning. **B** Burr hole placement. **C** Creation of a working channel. **D** Insertion of the endoscope. **E** Visualization and localization. **F** Resection or biopsy

- *Patient positioning* (Fig. 1A): The patient is placed in a supine position on the operating table. The skull is fixed using either a Mayfield clamp or a horseshoe headrest, slightly inclined to facilitate optimal surgical access.
- *Burr hole placement* (Fig. 1B): Depending on the location of the lesion within the third ventricle, a coronal burr hole is made for anterior lesions or a far frontal burr hole (up to 6 cm precoronal) is made for posterior lesions. The burr hole is carefully positioned using anatomical landmarks and imaging guidance to ensure accurate trajectory.
- *Creation of a working channel* (Fig. 1C): A small cortical incision is made at the burr hole site, and a trocar is inserted bimanually to create a working channel. The trocar is gradually advanced to the level of the ventricle under continuous monitoring to avoid injury to vital structures. Once the ventricle is reached, the holding arm of the system is fixed.
- *Insertion of the endoscope* (Fig. 1D): A rigid rod lens endoscope, such as the Karl Storz Hopkins® rod lens system (Karl Storz Endoskopie, Tübingen, Germany), with a suitable vision angle (e.g., 0°, 30°, or 70°) is carefully inserted through the working channel and advanced through the lateral ventricle towards the third ventricle. Angled optics provide direct visualization of the surgical field, enabling the surgeon to navigate within the ventricular system.
- *Visualization and localization* (Fig. 1E): The endoscope is maneuvered within the third ventricle to visualize the lesion and assess its precise location and anatomical relationships. The surgeon utilizes the different vision angles to optimize visualization and adapt to the specific configuration of the lesion.
- *Resection or biopsy* (Fig. 1F): Depending on the nature of the lesion, appropriate surgical maneuvers are performed. For cystic lesions, careful dissection and fenestration may be carried out using endoscopic bipolar or a laser to decompress and remove the cystic contents. For solid tumors, targeted biopsies or tumor resection may be performed using endoscopic forceps under direct visualization.
- *Hemostasis and closure*: The surgical assistant should contribute to the surgical success by constant irrigation through the working channel. It is of utmost importance to ensure an open outflow valve at the working channel. Otherwise, constant irrigation will lead to raised intracranial pressure with fatal consequences. Intraoperative bleedings can be handled by irrigation in most cases. The use of the endoscopic bipolar should be minimized to persisting bleedings from visualized vessels. Once the procedure is complete, the endoscope is carefully removed, and the working channel is gently withdrawn. By retracting the endoscope, the fornix and the entire trajectory are carefully inspected looking for contusions. The incision is closed using absorbable sutures or tissue adhesive.
- *Postoperative care*: Following surgery, the patient is closely monitored in the intermediate care unit or intensive care unit. Imaging studies, such as postoperative CT or MRI, are performed to assess the extent of resection and the absence of complications. Postoperative care includes pain management, monitoring for CSF leaks, and appropriate follow-up to evaluate clinical outcomes.

## Results

### General results of the study population

Since December 2010 until today, a total of 578 endoscopic transforaminal surgeries within the third ventricle were performed at the department of the authors. One-hundred thirty-two of 578 surgeries were conducted in pediatric patients. Excluding pure CSF restoration surgeries, a total of 24 surgeries for pediatric lesions within the third ventricle were included in the final analysis. The mean age at the time of surgery was 7.6 years (± 6.1 years). The most frequently performed procedures were cyst fenestrations or resections (13 cases), followed by tumor resections (4 cases), and inspections and restorations of the CSF pathway and tumor biopsies (7 cases total). Additional procedures included endoscopic third ventriculostomy (ETV) in 9 cases and the placement of a ventricular catheter (VP shunt or Rickham reservoir) in 12 cases. Two cases (8.3%) involved initial planning for a second approach to achieve gross-total tumor resection. All procedures were successfully completed without the need for premature termination nor conversion to an open microsurgical procedure with or without endoscopic assistance. Postoperative pneumocephalus occurred in 10 (41.7%) cases, but no emergency postoperative re-interventions were required. Self-limiting episodes of nausea and vomiting were seen in 14 cases (58.3%) postoperatively. One case (4.1%) needed temporary endocrinologic substitution for syndrome of inadequate antidiuretic hormone secretion (SIADH). There was no postoperative hemorrhage, no infection, nor perioperative mortality.

### Exemplary case 1

**Fig. 2:**
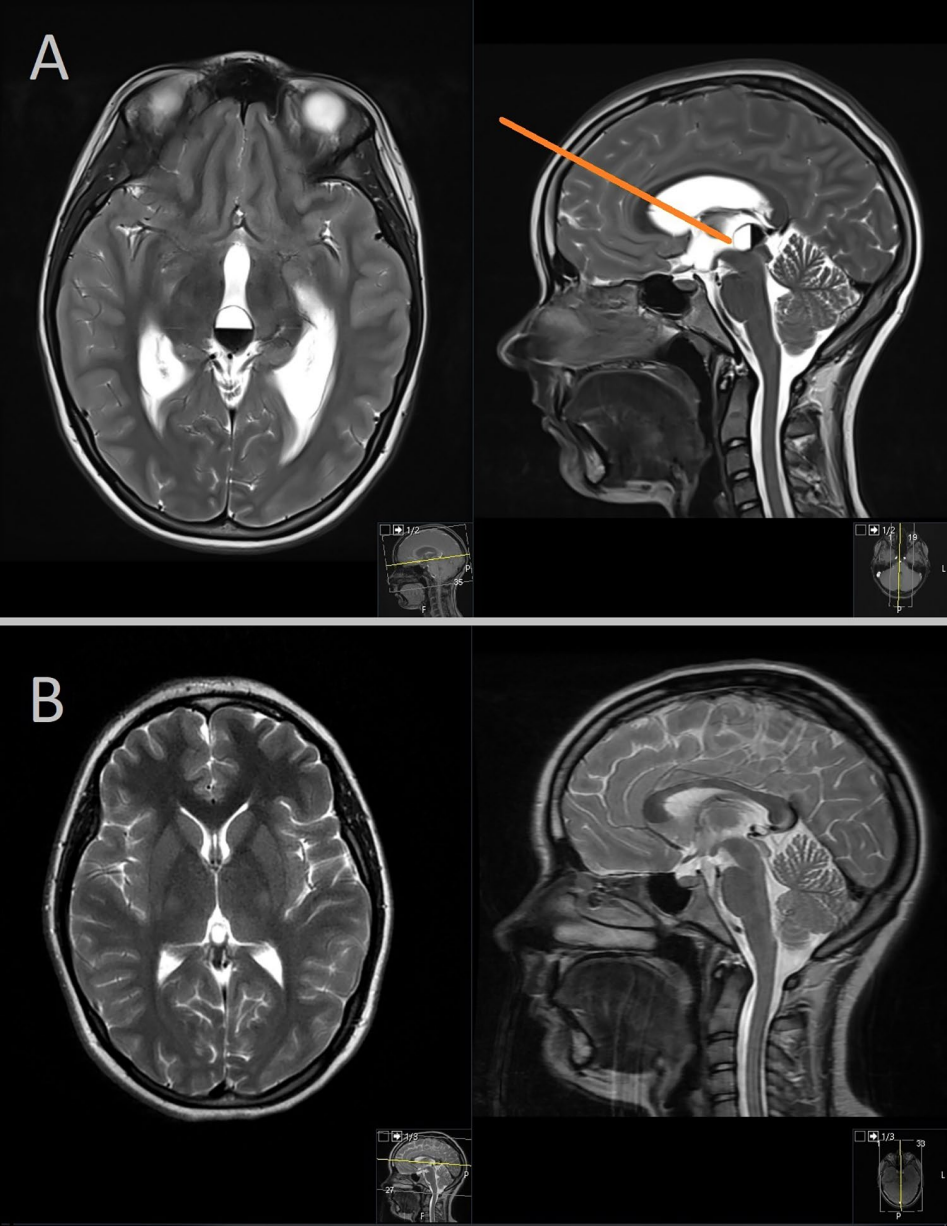
**A** Far frontal burr hole created to achieve the optimal trajectory towards the lesion. **B** Follow-up imaging demonstrating no evidence of lesion recurrence

The first case is a 14-year-old girl with therapy refractory headaches who underwent surgical intervention for a cystic lesion located within the posterior part of the third ventricle. Utilizing the endoscopic transventricular transforaminal approach, a far frontal burr hole was created to achieve the optimal trajectory towards the lesion (Fig. 2A). During the procedure, complete resection of the colloid cyst was successfully achieved. Postoperative care was uneventful, and the patient remained asymptomatic. Follow-up imaging demonstrated no evidence of lesion recurrence (Fig. 2B).

### Exemplary case 2

**Fig. 3:**
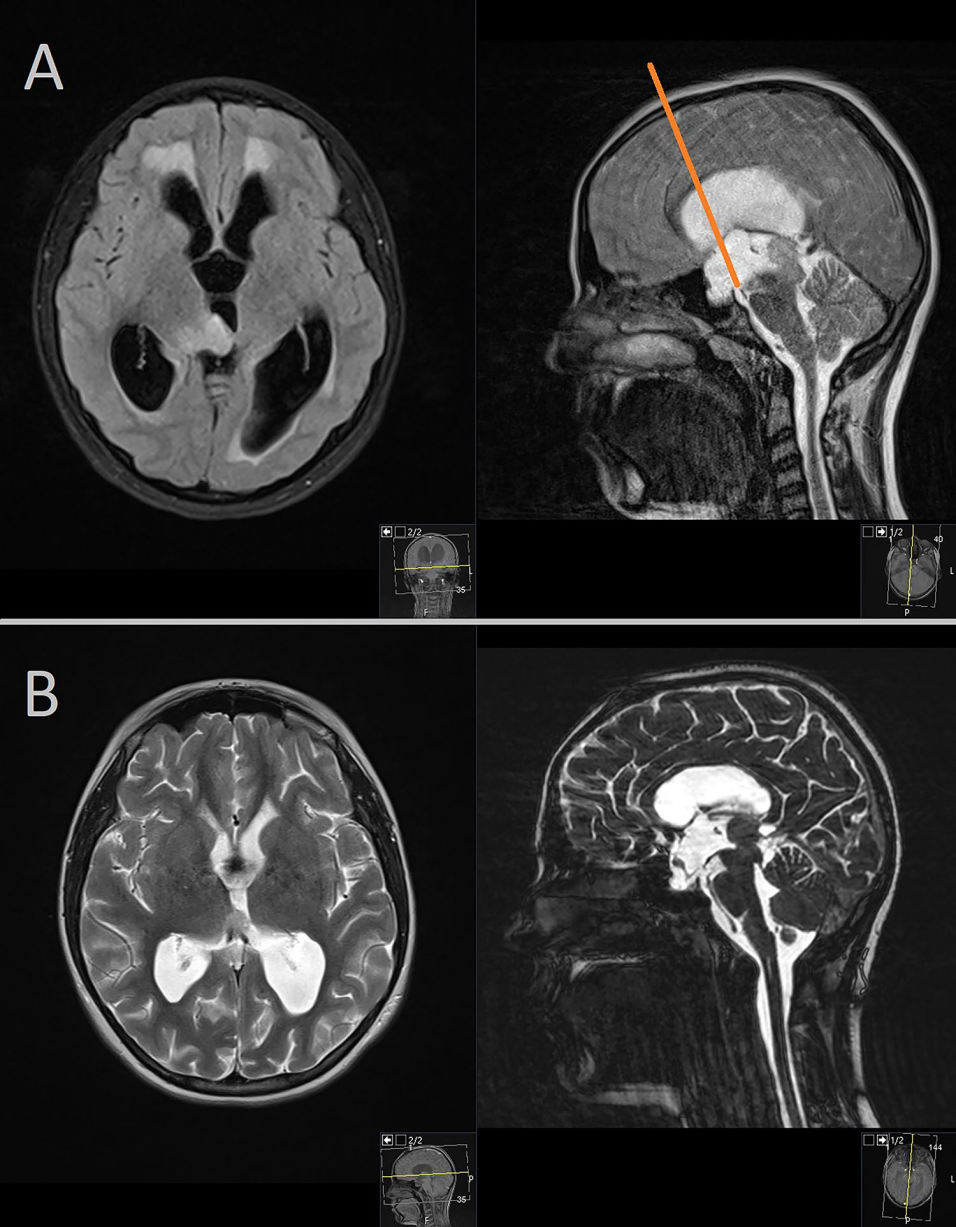
**A** MRI imaging revealing tumorous mass within the right thalamus and third ventricle. **B** Additional oncological treatment and follow-up MRIs performed postoperatively

The second case is a 6-year-old girl presenting with headaches and nausea, who underwent MRI imaging revealing a tumorous mass within the right thalamus and third ventricle, leading to aqueductal compression and subsequent hydrocephalus (Fig. 3A). Following an interdisciplinary oncologic conference, a surgical biopsy was recommended to establish an accurate histopathological diagnosis and guide further treatment. The patient underwent an endoscopic transventricular transforaminal approach, which allowed for both tumor biopsy and subsequent endoscopic third ventriculostomy (ETV) to restore the cerebrospinal fluid (CSF) pathway. The postoperative course was uneventful, with no complications observed. Histopathological analysis confirmed a low-grade glioma (WHO Grade I). The patient received additional oncological treatment, and follow-up MRIs were performed up to 4 years postoperatively (Fig. 3B), revealing good tumor control before the loss of follow-up due to the family’s relocation to another city.

### Exemplary case 3

**Fig. 4:**
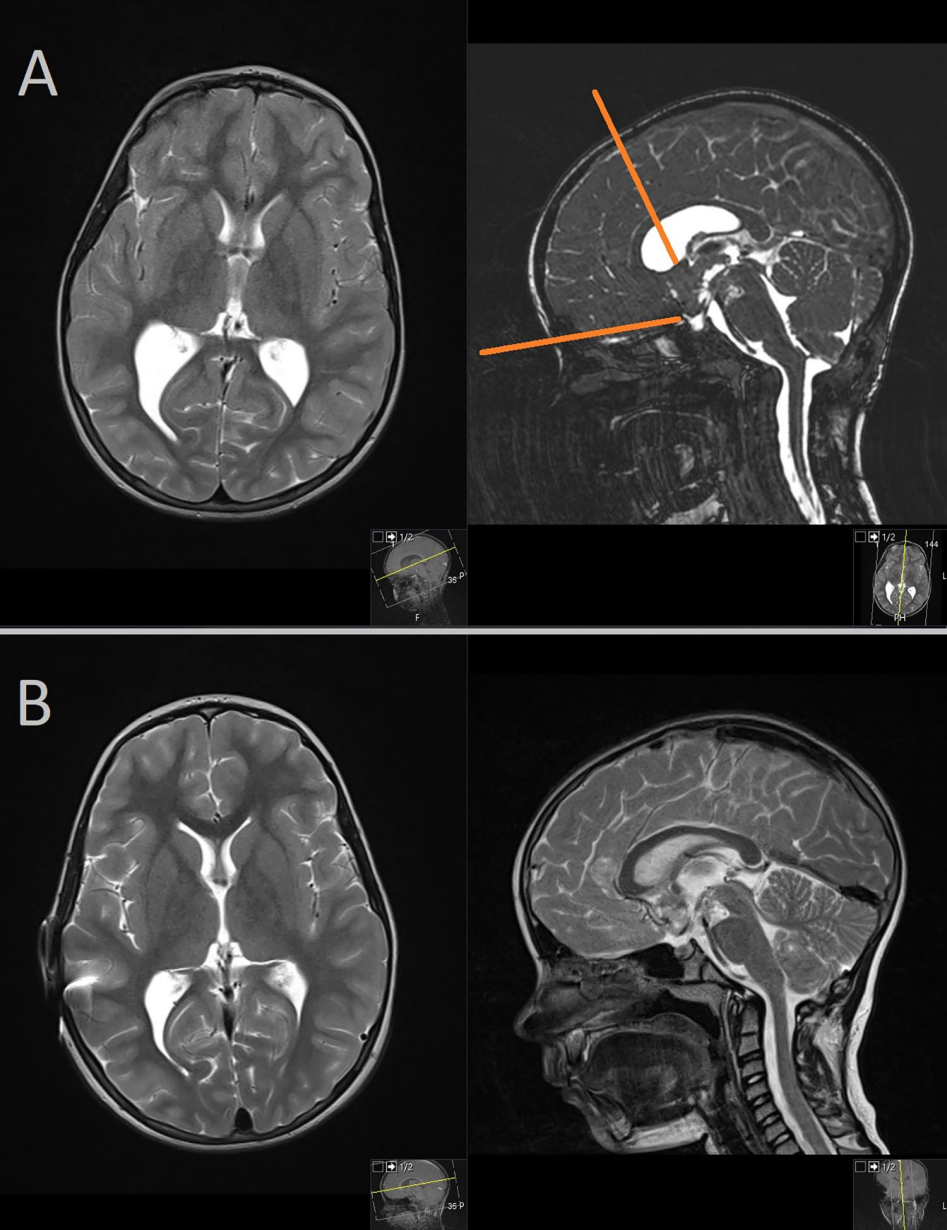
**A** MRI examination revealing a third ventricular tumor with frontobasal extension. **B** Radiological imaging demonstrating good tumor control without signs of progression

The third case is a 16-year-old girl who presented with headaches and vision deterioration. MRI examination revealed a third ventricular tumor with frontobasal extension, causing compression of the optic chiasm (Fig. 4A). Preoperative evaluation of the MRI findings indicated that the tumor could not be fully resected using a transventricular transforaminal approach alone. Consequently, a two-approach strategy was planned, involving the resection of the posterior part of the tumor via a transventricular approach and the resection of the more frontal part of the tumor via a supraorbital translaminar approach. Both surgical procedures were performed during a single session. Intraoperative assessment revealed that approximately 90% of the tumor was resectable. The postoperative course was uneventful, and histopathological analysis confirmed a diagnosis of pilomyxoid astrocytoma (WHO Grade I). The patient underwent further oncological treatment and underwent regular radiologic follow-up. Postoperatively, the patient experienced recovery of vision and resolution of headaches. At the 2-year follow-up, radiological imaging demonstrated good tumor control without signs of progression (Fig. 4B).

## Discussion

The endoscopic transventricular transforaminal approach has emerged as a valuable surgical technique for addressing various lesions within the third ventricle. The presented study supports the efficacy and versatility of this approach also in pediatric patients. The authors observed successful outcomes in all 24 procedures performed, including cyst fenestrations or resections, inspections and restorations of CSF pathway, tumor biopsies, and tumor resections. These findings highlight the applicability of the endoscopic transventricular approach for a range of procedures within the third ventricle.

The key to success in utilizing the endoscopic transventricular transforaminal approach lies in meticulous indication setting and planning. The precise positioning of the burr hole according to the location of the lesion within the third ventricle is critical. This experience aligns with the findings of the previous studies of Schroeder and Gaab [24, 27, 28] emphasizing the importance of accurate preoperative imaging and careful surgical planning to optimize the trajectory and minimize risks.

While the endoscopic transventricular approach is effective for most lesions within the third ventricle, there are instances where alternative approaches or a combination of approaches may be necessary. Large tumors located in the suprasellar region or far posterior lesions within the pineal region, for example, may require a different surgical strategy due to their unique anatomical challenges [6, 14, 29–31]. It is crucial for surgeons to recognize the limitations of the transventricular approach and consider alternative techniques when appropriate.

The successful execution of the endoscopic transventricular transforaminal approach heavily relies on the surgical experience and expertise of the operating surgeon. Proficiency in endoscopic techniques and maneuvering the endoscopes, especially within the narrow confines of the third ventricle, requires extensive training and a steep learning curve [27, 32, 33]. Continued education and exposure to a diverse range of cases are essential for achieving optimal outcomes with this operative technique.

Intraoperative troubleshooting is an integral aspect of the endoscopic transventricular approach. Bleeding complications and visual loss can occur during the procedure, necessitating prompt management. Constant irrigation, often for prolonged durations, has been effective in managing bleeding and preserving sufficient visualization [9, 34]. However, if visual impairment persists, a switch to microsurgical techniques should be considered to ensure patient safety and satisfactory outcomes [32]. A noteworthy technique that can be employed for specific situations of massive intraoperative bleedings and complete loss of vision is the “dry field technique,” as described by Oertel et al. [34]. This technique involves the suction of the entire CSF to maintain a clear field of vision and facilitate precise surgical maneuvers. The incorporation of the dry field technique in appropriate cases can enhance the safety and efficacy of the endoscopic transventricular approach.

In the pediatric population, cystic lesions are more prevalent than solid tumors within the third ventricle. The endoscopic transventricular transforaminal approach offers an effective treatment option for cystic lesions, allowing for fenestration or resection and subsequent restoration of CSF pathways [32]. Furthermore, the possibility of concurrent CSF pathway reconstruction via ETV through the same approach is a significant advantage when considering tumor surgery in children. ETV has proven to be a valuable adjunctive procedure in cases where CSF diversion is required, reducing the need for additional surgeries such as ventriculoperitoneal (VP) shunting [17, 35–37]. A subsequent minimization of potential complications associated with traditional CSF diversion procedures, such as infection, mechanical failure, and the need for revisions, may contribute to favorable long-term clinical outcomes.

## Conclusion

In conclusion, the endoscopic transventricular transforaminal approach is a valuable technique for managing third ventricle lesions in pediatric patients, offering a wide range of procedures. However, its limitations should be recognized, and alternative strategies should be considered for specific cases. The expertise of the surgical team, meticulous planning, and intraoperative troubleshooting techniques are crucial for optimal outcomes. Continued research and collaboration will contribute to further refining and expanding the applications of this approach.

## Data Availability

Anonymous data of patients are available upon request from the corresponding author.

## Abbreviations

CSF: Cerebrospinal fluid
ETV: Endoscopic third ventriculostomy
